# Therapeutic Effect of Murine Bone Marrow-Derived Mesenchymal Stromal/Stem Cells and Human Placental Extract on Testicular Toxicity Resulting from Doxorubicin in Rats

**DOI:** 10.1155/2021/9979670

**Published:** 2021-08-06

**Authors:** Mervat Ahmed AbdRabou, Ahmed B. M. Mehany, Islam M. Farrag, Amany Belal, Othman F. Abdelzaher, Abdou El-Sharkawy, Asmaa M. Abd El-Azez, Salah M. EL-Sharkawy, Manal H. Al Badawi

**Affiliations:** ^1^Biology Department, College of Science, Jouf University, P.O. Box 2014, Sakaka, Saudi Arabia; ^2^Zoology Department Faculty of Science, Al-Azhar University, Cairo, Egypt; ^3^Forensic Medicine and Clinical Toxicology, Faculty of Medicine (Girls), Al-Azhar University, Cairo, Egypt; ^4^Department of Pharmaceutical Chemistry, College of Pharmacy, Taif University, P.O. Box 11099, Taif 21944, Saudi Arabia; ^5^Department of Anatomy, Faculty of Medicine, Al-Azhar University, Cairo, Egypt; ^6^Department of Anatomy, College of Medicine, Jouf University, Saudi Arabia; ^7^Zoology Department, Faculty of Science (Girls), Al-Azhar University, Cairo, Egypt; ^8^Department of Anatomy, Faculty of Medicine, Helwan University, Helwan, Egypt

## Abstract

Oncotherapeutics like doxorubicin can affect male gonads; as a result, it leads to infertility. This work was conducted to demonstrate the toxic effects of doxorubicin on testes of male albino rats. Fifty male albino rats aged 5-7 weeks were used in this study. The animals were randomly separated into 5 sets (each set containing ten rats). Group I received saline (i.p.) for 4 weeks. Group II was given doxorubicin (DOX), 5 mg/kg BW (i.p.) once/week for 4 weeks. Groups III and IV were treated in the same way as the DOX group, left for one week without medication, and then injected with mesenchymal stromal cells (MSCs) or human placental extract (HPE) therapy in a single dose of 5 × 10^6^ in 200 ml PRP/week or 40 *μ*l placental extract for 4 weeks via the caudal vein. Group V rats were treated in the same way as the DOX group also, left for one week without medication, and then injected with MSC+HPE. A significant decrease in serum testosterone, FSH, and LH levels was observed in rats treated with DOX compared to the control group. A significant elevation was recorded in rats treated with DOX+MSC or DOX+HPE when compared with the DOX group only. Rats that were given MSC+HPE after DOX intoxication showed a significant increase in hormone levels when compared to rats treated with either MSC or HPE. Light and electron microscopic examinations revealed that DOX intoxication initiated degenerative and necrotic changes in seminiferous tubules associated with partial or complete cessation of spermatogenesis. These effects were reversed by the effect of MSC or HPE. Coadministration of MSC and HPE even showed further improvement. Finally, we can say that doxorubicin has a deleterious impact on rat testes; however, therapeutic effects can be induced through MSC and/or HPE administration.

## 1. Introduction

Oncotherapy includes systromalatic administration of agents that have cytotoxicity on cells [1]. Most of these agents cannot differentiate between the healthy and malignant cells; they accumulate in normal tissues resulting in cytotoxicities [2]. In recent years, temporary or permanent effects on male fertility are resulting from oncotherapeutic agents, such as surgery, radiation therapy, and chemotherapy [3]. The World Health Organization (WHO) considers marriage to be infertile if pregnancy did not occur after 12 months of intercourse [4]. One of the most serious effects of chemotherapy is testicular damage, as the testes are considered nontargeted healthy tissues that are affected due to their high proliferation activity [5]. Doxorubicin (DOX) (also known as Adriamycin) is one of the anthracycline antibiotics that have the ability to treat many types of malignant tumors, including breast cancer, bladder cancer, ovarian cancer, and uterine cancer [6]. Although DOX has shown effectiveness in the treatment of many malignant tumors, it has been reported to induce toxicity [7] such as cardiac, lung, and skeletal muscle dysfunction, nephrotoxicity, hepatotoxicity, and testicular toxicity [8]. On the other hand, DOX was reported to cause injury to the testicular germinal epithelium and reduction in sperm proliferation, quality, structure integrity, and motility average with increased cell death in spermatogonia and spermatocytes [9]. These effects are male concerns in adults suffering from cancer with highly curative cases [10]. Therefore, it is necessary to study the toxicity of doxorubicin on the whole body structure of the male reproductive system, particularly in testes where sperm proliferation can occur [11]. DOX is a drug that attacks cells in the cell cycle; it causes inhibition of the topoisomerase II enzyme, which is responsible for DNA strand intercalation [12]. Recent studies suggested that doxorubicin can induce changes causing abnormal chromosomes and cancer cells with many toxic effects on tissues such as testes [13]. The testicular injury mechanism of DOX is not fully understood; however, reports from previous studies suggested strongly oxidative stress, lipid peroxidation, and cellular apoptosis [14]. Previous studies have reported that DOX can produce many reactive oxygen species (ROS) by generating free radicals that can cause molecular changes [15]. In addition, DOX inhibits the neutralizing enzymes of ROS that naturally exist in the organs [16]. The disparity of the oxidant-antioxidant system causes tissue damage due to lipid peroxidation and tissue protein oxidation [17], retarded testicular growth, and disrupted spermatogenesis, which ultimately lead to infertility for males [14]. Previous studies reported that antioxidant intake enhanced the quality of the semen profile in infertile men [15]. Therefore, the coadministration of DOX with a potent antioxidant may be a convenient procedure to reduce its toxic effects. Mesenchymal stromal cells (MSCs) are a type of cells developed from various places of the body such as the bone marrow, fat tissue, and amniotic membrane [16]. Male infertility caused by the destruction of germ cells can be treated using cell transplantation [17, 18]; in addition to surgical operations, nowadays, there is a good strategy in treatment using stem cell therapy [19]. MSCs are undifferentiated, unspecialized cells which are renewed through cell division for long periods and can multiply, change, and progress into another type of cells with specific functions [20]. MSCs have developed and progressed rapidly for cell replacement therapy in recent years [21]. MSCs have a migratory capacity that can move to sites of defect when transplanted [22]. MSCs can develop into new cells of the injured organ and behave as an integrated element of the organ, so they could be viewed as clinically regenerative cell types for a lot of diseases and can help in cell treatment [23]. Several studies have confirmed that the possible treatment effects of stromal cells include adhesion with cells of the organ, immune modulation, and paracrine mechanisms that are triggered by trophic mediators [24]. Also, it helps in decreasing fibrosis, apoptosis, and intensification of angiogenesis [25]. Stromal cell biology concentrates on the protection roles of stromal cells to restore organs from ROS deleterious toxicity [26]. Several studies in rats have reported that MSCs survive in the testes, move to the basement membrane of the seminiferous tubules, and multiply and give additional germ cells from primary spermatocytes, spermatids, and spermatozoids in some seminiferous tubules of the rats [27]. The reversal of damage to testicular tissue by bone marrow-derived mesenchymal stromal cells (BM-MSCs) has been documented in experimental animals treated with busulfan [28]. MSCs can be located on the basement membrane of seminiferous tubules and improved the architecture of testicular cells [29]. This improvement in the action of MSCs refers to the release of growth factors and cytokines by stromal cells, causing proliferation activities [30]. The placenta is an essential mammalian organ during pregnancy. Its purpose is to provide growth requirements to the fetus. It can also adjust maternal immune responses and contain many bioactive materials, such as peptides, hormones, growth factors, and cytokines [31]. Human placental extract (HPE) is playing an important role in wounds and inflammation recovery [32]. Recent research studies have recorded that HPE has many bioactive components, such as polydeoxyribonucleotides, RNA, DNA, amino acids, and enzymes [33]. In addition, HPE possesses activities such as anti-inflammation, antipigmentation, antisunburn, antianaphylaxis, and antimutagenesis [34]. Also, it has been reported to inhibit diseases such as atherosclerosis and edema caused by oxidative stress [35]. The human placenta and its extracts have biological effects, such as modulating immune responses, protecting and regenerating hepatocytes, and regulating the hormonal balance of females, neurological effects on the activity of the brain, and anticoagulation and acceleration of wound healing [36]. HPE has been confirmed to treat and improve liver function in many Asian patients [37]. The current work was conducted to investigate the therapeutic effects of MSC and HPE versus DOX-stimulated apoptosis in testicular tissue.

## 2. Materials and Methods

### 2.1. Animals

Male albino rats (200-220 g) were 5-7 weeks old (mature) and were obtained from the Laboratory of Schistosome Biological Supply Program (SBSP), Theodor Bilharz Research Institute. The study was conducted at the Stem Cell Research Laboratory at Faculty of Science, Al-Azhar University, Nasr City, Cairo, Egypt. Animals were housed in animal houses under controlled conditions (12 hrs light) at room temperature and were fed a standard diet. Animal care and housing are in accordance with the Guide for Care and Use of Laboratory Animals.

### 2.2. Chemicals and Reagents

Doxorubicin is a red-orange powder; its brand name is Adriamycin (chemical formula: C_27_H_29_NO_11_, molecular weight: 543.525 g/mol), purchased from Sigma-Aldrich Corporation (St. Louis, MO, USA).

Human placental extract was purchased from Sigma (placental extract was concordant with those for Laennec, a drug of placental extracts for liver dysfunction that has been approved by the Ministry of Health, Labour and Welfare of Japan). Cyclo-trans-4-L-hydroxyprolyl-L-serine (also called JBP485) is isolated from hydrolysates of placental extract.

Other chemicals and reagents used in this study are as follows: thiopental sodium 500 mg (Sigmatec Pharmaceutical Industries); sodium heparin; Dulbecco's low glucose modified Eagle's medium (HyClone, Logan, UT, USA); antibiotic-antimycotic (Gibco, Grand Island, NY, USA); Ficoll-Paque 1.077 g/mL (GE Biosciences, Piscataway, NJ, USA); Minimum Essential Media (MEM) (Gibco/BRL, Gaithersburg, MD, USA); penicillin 100 mg/mL of streptomycin 100 units/mL (Atlanta Biologicals, Atlanta, GA, USA); L-glutamine (Gibco/BRL); phosphate-buffered saline (PBS); trypsin/EDTA; trypan blue; and NaCl 0.9% (El-Nasr Company, Egypt).

### 2.3. MSC Preparation

MSCs were isolated according to a protocol modified from [38]. Flushing was used to extract bone marrow from the tibiae and femurs of 8-week-old male Sprague Dawley albino rats with Dulbecco's modified Eagle's medium (DMEM, Gibco/BRL); flushing was continued until bones appear white; red clumps of cells were dispersed by crossing the cell suspension via the 21-gauge needle until no more clumps are seen. The supernatant was collected and passed via a 70 *μ*m cell strainer. In a cell strainer, the fragments of bone were washed by mixing them with an added 10 ml of buffer and letting the remains relax for 3-4 minutes. Filter wash via 70 *μ*m sieves that connect to the cells previously collected.

The cell suspension was homogenized by pipetting up and down and then centrifuged at 300 × *g* (~1200 rpm) and 4°C for 10 min with the brake on; the supernatant was discarded, and the pellet was resuspended in 10 mL of DMEM.

### 2.4. Flow Cytometry for Characterization of MSCs

The cells were subcultured twice to have a number of cells for characteristics and cell therapy. Cells in passage no. 3 were collected and counted using a hemocytometer. They were cryopreserved through the conventional method by dimethyl sulfoxide until use. After that, the cells in a fresh medium were cultured and subcultured. The cultured cells were suspended in cold DPBS at the dilution of 10^6^ cells/ml. Incubation with primary antibodies (CD45, CD90, CD105, and CD166) was followed by exposure to the secondary conjugated antibody at 4°C for 30 min, and the complex was analyzed by flow cytometry.

### 2.5. Experimental Animal Design

Fifty male rats were divided into five groups consisting of ten animals in each group. Group I (control group) rats received 0.1 ml/day saline (i.p.) for 4 weeks. Group II (DOX group) received 5 mg/kg BW (i.p.) of doxorubicin (DOX) once/week for four weeks to reach a cumulative dose of 20 mg/kg [39]. Group III (DOX+MSC group) rats were treated the same as the DOX group, left for one week without medication, and then injected with MSC therapy in a single dose of 5 × 10^6^ in 200 ml PRP/week for 4 weeks via the caudal vein [40]. Group IV (DOX+HPE group) rats were treated the same as the DOX group, left for one week without medication, and then injected with human placental extract (HPE) in a single dose of 40 *μ*l placental extract/week for 4 weeks via the caudal vein [41]. Group V (DOX+MSC+HPE group) rats were treated the same as the DOX group, left for one week without medication, and then injected with MSC (5 × 10^6^) in 200 *μ*l PRP+placental extract (40 *μ*l) in a single dose/week for 4 weeks via the caudal vein. The treatment protocol was designed to start MSC and HPE one week after the last DOX injection to ensure adequate elimination of DOX from rat bodies so as to not damage introduced stromal cells with its cytotoxic action [42, 43]. The human placental extract (Laennec) rat dose was calculated by converting the dose taken by cirrhotic patients for liver regeneration (2 ml/70 kg) by using conversion factors [44].

### 2.6. Analysis of Blood Samples

Blood samples (5 ml/rat) were withdrawn by cannulation via the jugular vein by adapting the technique of [45]. Collected blood was used to measure testosterone hormone levels in serum according to [46] and follicle-stimulating hormone and luteinizing hormone levels in serum according to [47, 48], respectively. Hormones were measured using (Human) ELISA diagnostic kits (Catalog Number KA0236).

### 2.7. Testicular Hormone Assay

Serum samples were stored at −70°C until use. Levels of LH, FSH, and T were measured in serum using RIA kits according to the manufacturer's protocol.

### 2.8. Light Microscopic Examination

An automated tissue processor was used to process the formalin-fixed testes. The processing began with two steps (fixation and dehydration): fixation: tissues were immersed in 10% buffered formalin for 48 hours, then washed in distilled water for 30 minutes to remove the fixative; and dehydration: the tissues were dehydrated by putting them through a graded series of alcohols (70%, 90%, and 100%). The tissue was first subjected to 70% alcohol for 120 minutes, then to 90% alcohol for 90 minutes, and finally to absolute alcohol, each for one hour. After dehydration, the samples were cleared in several xylene changes. It consisted of immersing the fabric in a mixture of 50% alcohol and 50% xylene for one hour, followed by an hour and a half of pure xylene. The samples were then embedded and masked after being impregnated with molten paraffin wax. Paraffin sections (4-5 *μ*m) were stained with hematoxylin and eosin [49, 50]. The stained sections were examined for circulatory disorders, inflammation, apoptosis, necrosis, degeneration, and any other pathological alternations in the examined tissues.

### 2.9. Histomorphometric Analysis of Testes

Histomorphometric analysis was done using “ImageJ®” (an open-source image processing software program designed for scientific multidimensional images).

### 2.10. Electron Microscopic Investigations

The fresh tissue was primarily fixed by immersion in 2.5% glutaraldehyde, then secondarily fixed by immersion in 1% osmium tetroxide, and then washed in 0.1 M cacodylate buffer. Fixed and washed samples were dehydrated by increasing alcohol content. The absolute alcohol was replaced by propylene oxide. Dehydrated samples were penetrated with the resin mixture via a tiered series in glass vials with polypropylene caps and then transported to an oven.

The mounting blocks were trimmed with razor blades using an ultramicrotome. Semithin sections (1 *μ*m thick) were cut with glass knives and stained with toluidine blue. Ultrathin sections (50-80 nm thick) were cut with glass knives, and the sections floated on the water surface, which was recorded on copper grids. The sections were double-stained in uranyl acetate then by lead citrate. Stained sections on grids were washed with 0.02 N NaOH followed by distilled water and left on filter paper in a petri dish [51] prior to examination with a JEOL 1010 Transmission Electron Microscope at the Regional Center for Mycology and Biotechnology (RCMB), Al-Azhar University.

### 2.11. Statistical Analysis

Data of the seminiferous ductal diameter, seminiferous luminal diameter, seminiferous epithelial height, and interstitial space diameter in all groups were analyzed by one-way ANOVA (SPSS for Windows, version 11.5). *p* ≤ 0.05 was considered to be statistically significant.

## 3. Results

### 3.1. Flow Cytometry Analysis for MSCs

**Figure 1:**
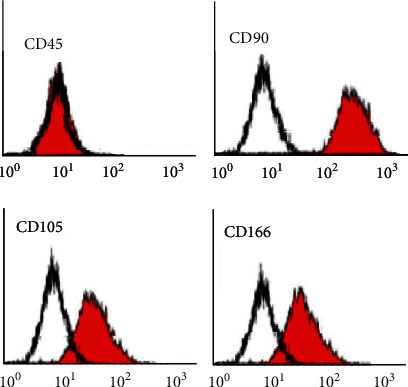
Flow cytometry for markers of MSCs: CD45, CD90, CD105, and CD166.

Cells after examination showed CD45 to be negative while CD90, CD105, and CD166 to be positive (98.76%, 96.27%, and 95.31%), respectively, as shown in Figure 1.

### 3.2. Testicular Hormone Results

**Figure 2:**
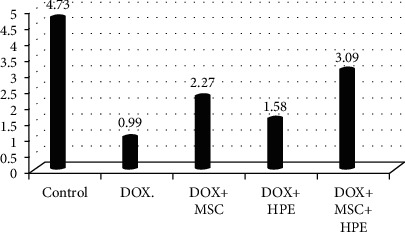
Serum testosterone (ng/ml) in adult male albino rats subjected to different treatment conditions.

**Figure 3:**
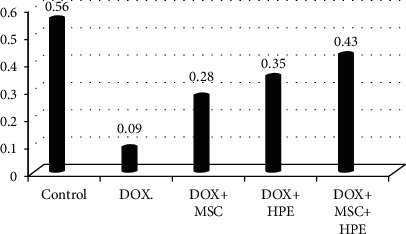
Serum FSH (mIU/ml) in adult male albino rats subjected to different treatment conditions.

**Figure 4:**
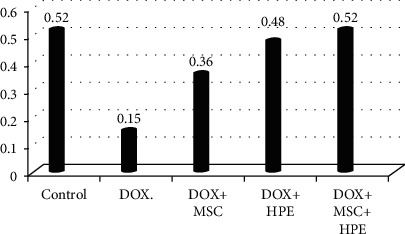
Serum LH (mIU/ml) in adult male albino rats subjected to different treatment conditions.

Table 1 records a significant decrease (*p* < 0.05) in serum testosterone, FSH, and LH levels in rats treated with DOX, compared to the control group. In contrast, rats treated with DOX+MSC and DOX+HPE showed a significant increase (*p* < 0.05) when compared with intoxicated groups. DOX+MSC+HPE-treated rats showed a significant increase (*p* < 0.05) as compared to rats treated with DOX in addition to MSC or HPE (Figures 2–4).

### 3.3. Light Microscopic Findings

**Figure 5:**
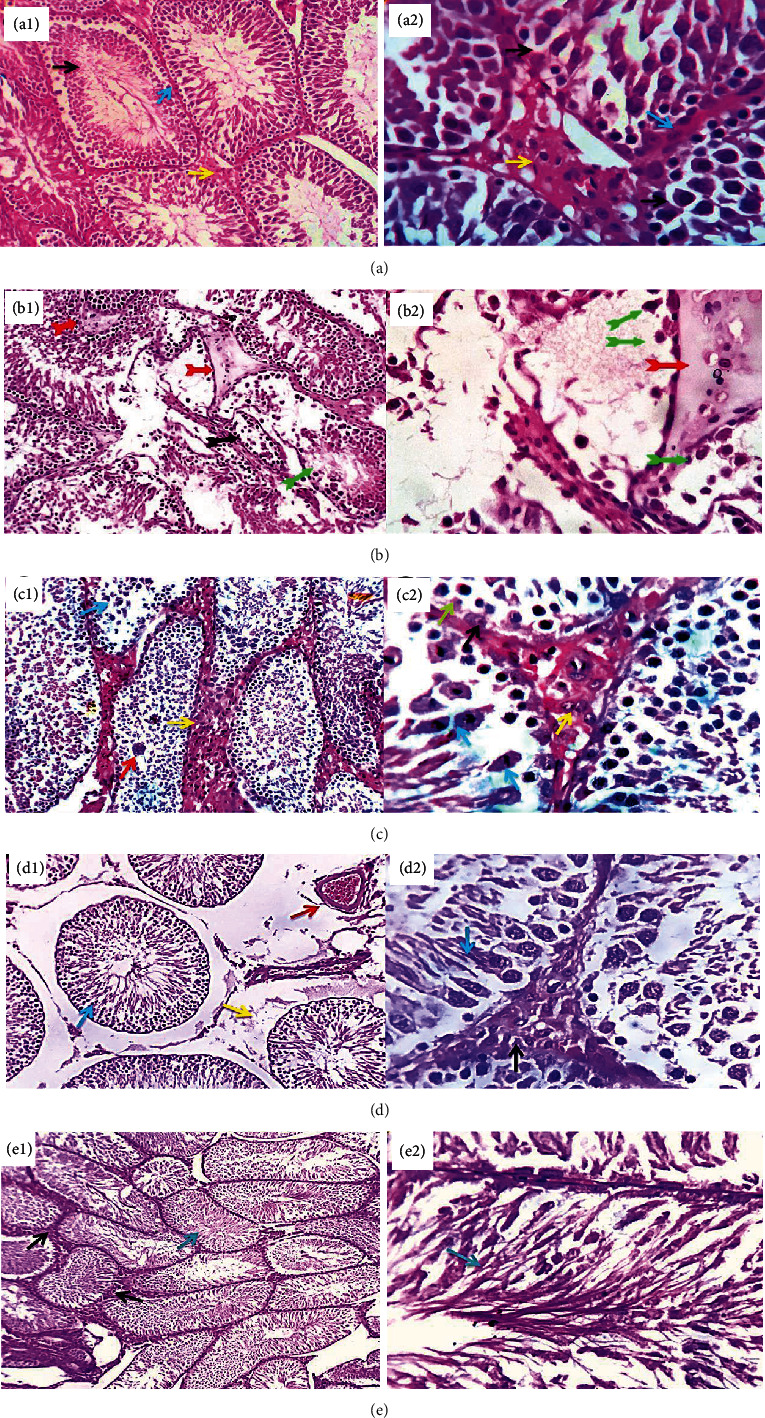
(a1, a2) Normal group showing apparently normal seminiferous tubules (blue arrows) and interstitial Leydig cells and the stroma (yellow arrows). (b1, b2) Doxorubicin group showing degenerative and necrotic changes in a moderate number of seminiferous tubules associated with partial or complete arrest of spermatogenesis (black and green arrows) with the spermatozoon-free lumina. The testicular blood vessels are congested (red arrow), and the interstitial tissue appeared edematous with loss of a large number of Leydig cells (brown arrow). (c1, c2) Doxorubicin+MSC-treated group showing functional seminiferous tubules with healthy actively dividing spermatocytes and fully matured spermatozoa. The dividing cells showing different steps of cellular division with hyperchromatic nuclei (blue arrows), sometimes with the formation of sperm giant cells (red arrow). A few germinal epithelial cells are degenerated or apoptotic (black and green arrows). The interstitial tissue was mildly thickened by proliferated Leydig cells and mild edema (yellow arrows). (d1, d2) Doxorubicin+HPE-treated group showing mild congestion of the testicular blood vessels (red arrow), interstitial edema with diminished Leydig cell population (yellow arrow), and partial atrophy of some seminiferous tubules (d1, blue arrow). The majority of the latter were healthy with active spermatogenesis and spermiogenesis (d2, blue arrow). The interstitial tissue and Leydig cells are apparently normal (black arrow). (e1, e2) DOX+MSC+HPE-treated group showing normal seminiferous tubules with active spermatogenesis and spermiogenesis with fully matured spermatozoa filling their lumina (blue arrows). Mild focal thickening of the interstitial tissue by proliferated Leydig cells is seen (black arrows). H&E ×200 (1) and ×400 (2).

DOX administration promoted degenerative and necrotic changes in a moderate number of seminiferous tubules associated with partial or complete arrest of spermatogenesis with the spermatozoon-free lumina. The testicular blood vessels were congested, and the interstitial tissue appeared edematous with loss of a large number of Leydig cells (Figure 5(b)) as compared to the control group (Figure 5(a)). These effects were ameliorated in rats treated with MSC or HPE after DOX intoxication (Figures 5(c) and 5(d)) with functional seminiferous tubules showing healthy actively dividing spermatocytes and fully matured spermatozoa; few germinal epithelial cells were degenerated or apoptotic. The interstitial tissue was mildly thickened by proliferated Leydig cells and mild edema as compared to rats treated with DOX alone. Coadministration of MSC and HPE after DOX intoxication significantly improved testicular histomorphological structure; the seminiferous tubules showed active spermatogenesis and spermiogenesis with fully matured spermatozoa filling their lumina. Mild focal thickening of the interstitial tissue by proliferated Leydig cells was encountered (Figure 5(e)).

**Figure 6:**
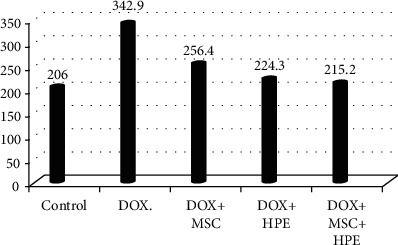
The seminiferous ductal diameter in adult male albino rats subjected to different treatment conditions.

**Figure 7:**
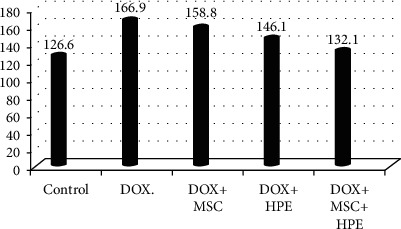
The seminiferous luminal diameter in adult male albino rats subjected to different treatment conditions.

**Figure 8:**
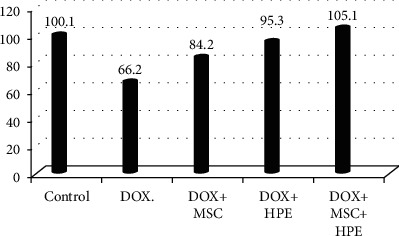
The seminiferous epithelial height in adult male albino rats subjected to different treatment conditions.

**Figure 9:**
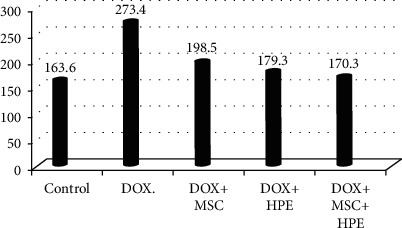
The interstitial space diameter in adult male albino rats subjected to different treatment conditions.

Table 2 illustrates the analysis of histomorphometric estimation and indicates that the seminiferous ductal diameter in G4 and G5 was less than that in the group treated with doxorubicin (G2), while rats with doxorubicin treatment (G2) showed a significant increase compared with the control group. There were significant differences in the seminiferous luminal diameter in the rat testes of G2, G3, and G4 (*p* < 0.05), while no significant indication for G5 when compared with the control group. However, the S. epithelial height in rats of G4 and G5 was not statistically different (*p* > 0.05) with the normal control group. But the seminiferous epithelial height in the group treated with doxorubicin (G2) showed a markedly significant decrease (*p* > 0.05) when compared to the control group (G1). Finally, the interstitial space diameter of the rats in G2 and G3 was significantly elevated compared to the control group (*p* < 0.05) (Figures 6–9).

### 3.4. Electron Microscopic Findings

**Figure 10:**
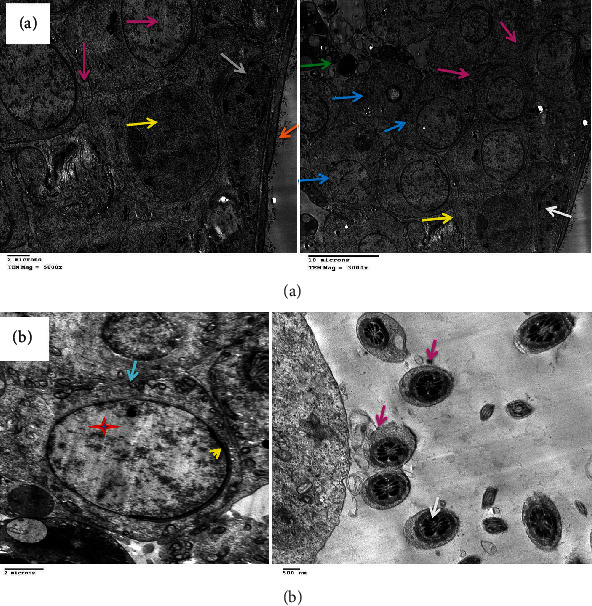
(a) Electron micrograph of testes of the DOX-treated group showing marked thickening of the basement membrane (orange arrows) and complete disappearance of spermatogonia with the presence of Sertoli cells (yellow stars), a few mitochondria (blue arrows), electron-dense bodies (dark blue arrows), apoptotic spermatocytes (red arrow), myofilaments (green arrows), and irregular vacuoles (black arrows). (b) Showing marked necrosis and apoptosis of deformed spermatozoa (red arrows), spermatocytes and spermatids (yellow and dark blue arrows), multiple macro- and microvesicles (light blue arrows), a few electron-dense bodies and mitochondria (orange and white arrows), early apoptotic spermatogonia and Sertoli cells (green and yellow stars), and shrunken myoepithelial cells (brown arrow).

**Figure 11:**
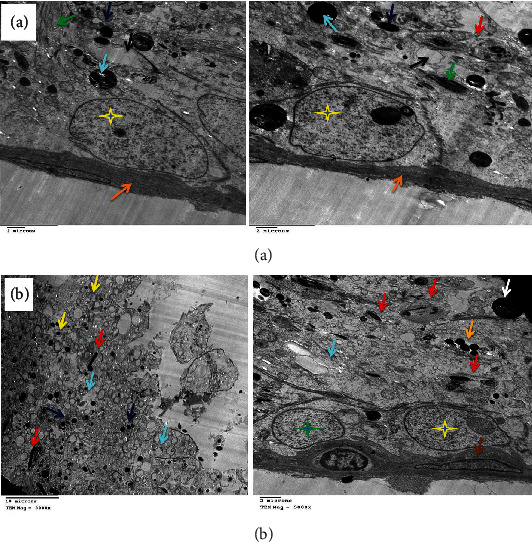
(a) Electron micrograph of testes of the control group showing normal spermatogenesis with normal morphology of the basement membrane (orange arrow), myoepithelial cells (white arrow), Sertoli cells (yellow arrow), spermatogonia (purple arrows), preleptotene primary spermatocytes (blue arrows), and early round spermatids (green arrow) showing some nuclear condensation. (b) Showing preleptotene primary spermatocytes (star), an intercellular bridge with membrane interdigitation (arrow), and early acrosome formation (arrowhead). Early round spermatids (purple arrows) with nuclear rosette shape condensation (white arrow) just prior to the cap phase of acrosome formation.

**Figure 12:**
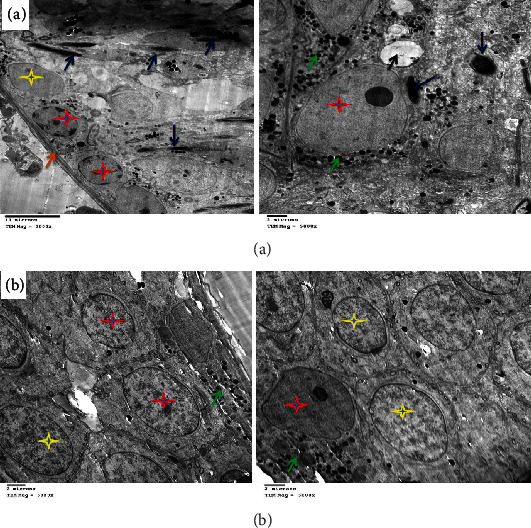
(a) Electron micrograph of testes of the DOX+MSC-treated group showing normal active spermatogonia (red stars), Sertoli cells with ill-distinct borders containing huge amount of mitochondria (yellow stars and light blue arrow), and dividing spermatocytes with intercellular electron-dense bodies (green arrows) and mild interstitial edematous granular material (dark blue arrows). (b) Showing normal basement membrane (orange arrow) and normal active spermatogonia containing a large number of mitochondria (red star and green arrows) and a moderate number of spermatozoa at different stages of maturation (dark blue arrows), surrounding Sertoli cells (yellow star). A macrovesicle is seen (black arrow).

**Figure 13:**
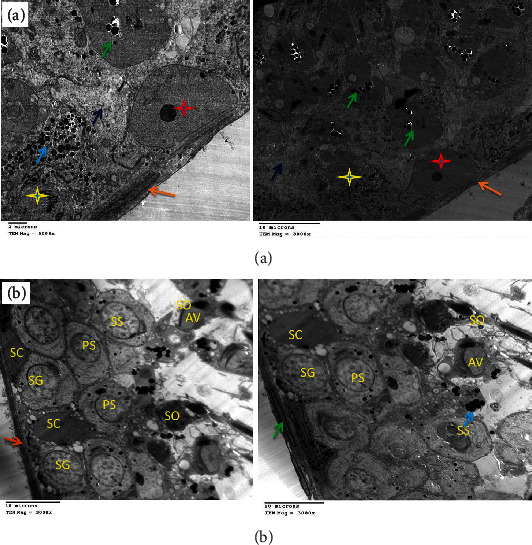
(a) Electron micrograph of testes of the DOX+HPE-treated group showing normal healthy spermatogonia (red stars) surrounded by a moderate number of mitochondria (green arrows) and dividing spermatocytes (yellow stars). (b) Showing a group of normal healthy spermatogonia (SG), Sertoli cells (SC), primary and secondary spermatocytes (PS, SS), and spermatozoa (SO) with acrosomal vesicle formation (AV), besides the normal basement membrane (orange arrow), myoepithelial cells (green arrow), and electron-dense bodies (blue arrow).

**Figure 14:**
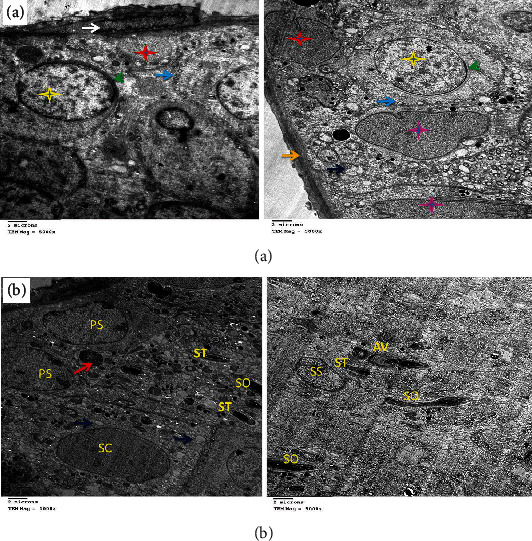
(a) Electron micrograph of testes of the DOX+MSC+HPE-treated group showing normal active spermatogonia (red star), preleptotene primary spermatocytes with early acrosome formation (yellow star and green arrowheads), and an intercellular bridge with membrane interdigitation and microtubular formation (blue arrows). Sertoli cells with many lipoid vesicles (purple stars and dark blue arrow), an intact basement membrane (orange arrow), and myoepithelial cells (white arrow). (b) Showing normal primary and secondary spermatocytes (PS, SS) and spermatids and spermatozoa (ST, SO) with acrosomal vesicle formation (AV), besides a moderate number of mitochondria (red arrow) and cytoplasmic microvesicles, especially in Sertoli cells (SC).

DOX administration affected testicular ultrastructure; testes demonstrated marked thickening of the basement membrane and complete disappearance of spermatogonia with the presence of Sertoli cells, a few mitochondria, electron-dense bodies, apoptotic spermatocytes, myofilaments, and irregular ill-distinct vacuoles. Marked necrosis and apoptosis of deformed spermatozoa, spermatocytes and spermatids, multiple macro- and microvesicles, a few electron-dense bodies and mitochondria, early apoptotic spermatogonia and Sertoli cells, and shrunken myoepithelial cells were characteristic. Some sections revealed the disorganized basement membrane of the seminiferous tubules, the marked necrosis and apoptosis of the spermatocytes and spermatids, and the presence of vesicles and electron-dense bodies (Figure 10) as compared to the control group (Figure 11). These effects were ameliorated in rats treated with MSC or HPE after DOX intoxication; control testes showed normal healthy spermatogonia surrounded by Sertoli cells, dividing primary and secondary spermatocytes, besides the normal basement membrane and electron-dense bodies; DOX+MSC-treated testes showed normal active spermatogonia containing a large number of mitochondria and a moderate number of spermatozoa at different stages of maturation, surrounding Sertoli cells; besides, the presence of cytoplasmic macrovesicles was also seen (Figures 12 and 13) as compared to DOX-intoxicated rats. Coadministration of MSC and HPE after DOX intoxication revealed marked restoration of the general testicular ultrastructure; sections revealed normal active spermatogonia, preleptotene primary spermatocytes with early acrosome formation, and an intercellular bridge with membrane interdigitation and microtubular formation. Sertoli cells with many lipoid vesicles, an intact basement membrane, and myoepithelial cells were seen. Normal electron morphology of primary and secondary spermatocytes and spermatids and spermatozoa with acrosomal vesicle formation, besides a moderate number of mitochondria and cytoplasmic microvesicles, especially in Sertoli cells, was also observed (Figure 14).

## 4. Discussion

DOX caused toxicity in germ cells by triggering oxidative stress and apoptosis [52]. Previous studies have reported that the damage in DNA caused by DOX leads to an increased synthesis of ROS and suppresses the activities of antioxidant enzymes that caused testicular dysfunctions [53]. In the present study, DOX-induced testicular toxicity is further determined by the histopathology findings in DOX-exposed animals. A high level of testosterone in testes is crucial for normal spermatogenesis, as well as more differences in the structure, morphology, and physiology of seminiferous tubules. Furthermore, electron microscopic examination showed that DOX administration affects testicular ultrastructure, such as the markedly thickened basement membrane and absent spermatogonia with the presence of Sertoli cells, a few mitochondria, electron-dense bodies, apoptotic spermatocytes, myofilaments, and irregular ill-distinct vacuoles. Also, marked necrosis and apoptosis of deformed spermatozoa, spermatocytes and spermatids, multiple macro- and microvesicles, and a few electron-dense bodies were shown. Combined administration of cancer chemotherapy together with potent antioxidant agents was proposed as an appropriate approach to counteract chemotherapeutic toxicity [54]. MSCs were reported to have widespread therapeutic uses, including wound healing and infertility, among others [55]. This study was conducted to investigate the DOX-induced testicular toxicity and the possible therapeutic role of MSC and HPE in rats. Obtained results showed there was a remarkable decrease in serum testosterone, LH, and FSH levels. This agreed with previous studies that DOX induced a significant decrease in serum testosterone, LH, and FSH, which affects spermatogenesis and structural morphology of seminiferous tubules [56]. In the current work, treatment with MSC or HPE reversed the levels of these hormones almost back to normal. This advance may be because MSCs can differentiate into germ cells. These observations are consistent with the results detected by researchers who reported that injected MSCs significantly restored serum testosterone, FSH, and LH hormones in rats [57]. The mechanisms involved in the restorative effects of MSCs on testicular function are due to the ability of transplanted MSCs to stimulate the production of substances capable of inhibiting ROS, cell death, inflammation, and mutagenic activities [58]. MSCs were able to differentiate into spermatocytes to release hormones [59]. On the other hand, MSCs play a role in restoring spermatogenesis by differentiating into sperm cells or maintaining spermatogonial stromal cells. Hence, MSCs could be an essential and powerful approach to treating infertility [58]. In the present study, animals treated with both MSC and HPE showed testicular hormones with nearly normal values, herewith throwing light on their combined therapeutic effect in DOX-induced testicular toxicity. Also, results illustrated that improvement in seminiferous tubule structure was associated with partial or complete arrest of spermatogenesis in DOX-treated rats on light microscopic examination. In the current work, the severity of testicular damage in rats treated with MSC or HPE was markedly improved when compared with the DOX group. These results are indicative of the therapeutic effect of MSC or HPE against doxorubicin gonadotoxicity. Earlier studies recorded that MSC treatment resulted in a rapid reversal of testicular injury and marked restoration of normal histological structure of testes after sterility induced by DOX [59]. The effect of MSCs is essentially happening through direct recovery of injured organs and cell development; this was manifested by the presence of MSCs in the interstitial connective tissue between the seminiferous tubules, and their morphology was like that of Leydig cells [60], or the effect is happening through indirect inducement of cell proliferation through paracrine signaling, which includes production of growth factors that help in interactions between cells [61]. Previous studies revealed that pretreatment with HPE reversed the extensive hepatic degeneration. Systromalatically, it has been reported that HPE has a variety of biological activities, such as anti-inflammatory [62] and antioxidant properties [63]. HPE can also produce proinflammatory cytokines and mediators. HPE has been reported to inhibit the production of nitric oxide, TNF-*α*, and cyclooxygenase-2 in lipopolysaccharide-stimulated RAW264.7 macrophages [64]. HPE showed both the antioxidant and anti-inflammatory activities in rats exposed to benzo[a]pyrene (BaP) [65]. In our study, animals treated with HPE showed reduced histological abnormalities, thereby highlighting its combined therapeutic roles in counteracting the cytotoxic injury induced by DOX.

In conclusion, the current study indicated that DOX carries the risk of severe testicular damage. The addition of MSC and HPE can provide a marked therapeutic effect which may draw attention that these agents can be used as a potential therapeutic adjuvant that could protect testicular tissue from DOX-induced testicular toxicity.

## Figures and Tables

**Table 1:** Serum testosterone (ng/ml), FSH (mIU/ml), and LH (mIU/ml) in adult male albino rats subjected to different treatment conditions.

Testicular hormones	Groups				
	Control	DOX	DOX+MSC	DOX+HPE	DOX+MSC+HPE
Testosterone (ng/ml)	4.73 ± 0.17^a^	0.99 ± 0.06^b^	2.27 ± 0.35^c^	1.58 ± 0.17^d^	3.09 ± 0.13^e^
FSH (mIU/ml)	0.56 ± 0.04^a^	0.09 ± 0.01^b^	0.28 ± 0.03^c^	0.35 ± 0.02^c^	0.43 ± 0.02^d^
LH. (mIU/ml)	0.52 ± 0.03^a,d^	0.15 ± 0.02^b^	0.36 ± 0.02^c^	0.48 ± 0.04^d^	0.52 ± 0.03^d^

Values are expressed as mean ± SD. *p* < 0.05. Each value represented means of 5 records ± S.E.^a,b,c,d,e^Comparison between all groups where the groups have the same letter means there is no significant difference, and comparison between all groups where the groups have different letters means there is a significant change. DOX: doxorubicin; MSC: mesenchymal stromal cell; HPE: human placental extract.

**Table 2:** The seminiferous ductal diameter, seminiferous luminal diameter, seminiferous epithelial height, and interstitial space diameter in adult male albino rats subjected to different treatment conditions.

Groups	S. ductal diameter (*μ*m)	S. luminal diameter (*μ*m)	S. epithelial height (*μ*m)	Interstitial space diameter (*μ*m)
Control (G1)	206 ± 6.4^a^	126.6 ± 1.2^a^	100.1 ± 7.3^a^	163.6 ± 2.5^a^
Doxorubicin (G2)	342.9 ± 30.6^b^	166.9 ± 4.8^b^	66.2 ± 2.2^b^	273.4 ± 9.9^b^
DOX+stem cell (G3)	256.4 ± 3.8^c^	158.8 ± 1.1^c^	84.2 ± 3.2^c^	198.5 ± 5.5^c^
DOX+placental extract (G4)	224.3 ± 7.5^a,c^	146.1 ± 1.7^d^	95.3 ± 1.9^a^	179.3 ± 2.6^a^
DOX+stem cell+placental extract (G5)	215.2 ± 4.5^a,c^	132.1 ± 1^a^	105.1 ± 2.1^a^	170.8 ± 3.9^a^

## Data Availability

All data are included in the online version of the manuscript.
